# Community Pharmacists’ Perceptions towards the Misuse and Abuse of Pregabalin: A Cross-Sectional Study from Aseer Region, Saudi Arabia

**DOI:** 10.3390/healthcare9101281

**Published:** 2021-09-28

**Authors:** Sultan M. Alshahrani, Khalid Orayj, Ali M. Alqahtani, Mubarak A. Algahtany

**Affiliations:** 1 Clinical Pharmacy Department, College of Pharmacy, King Khalid University, Abha 61441, Saudi Arabia; korayg@kku.edu.sa; 2 Pharmacology Department, College of Pharmacy, King Khalid University, Abha 62529, Saudi Arabia; amsfr@kku.edu.sa; 3 Division of Neurosurgery, Department of Surgery, College of Medicine, King Khalid University, Abha 62512-2291, Saudi Arabia; mbalgahtany@kku.edu.sa

**Keywords:** pregabalin, abuse, community pharmacists, Saudi Arabia, pain

## Abstract

Pregabalin is a first-line therapy for neuropathic pain and for chronic pain. It has abuse potential. This study was conducted to assess community pharmacists’ perceptions towards pregabalin abuse and misuse in the Aseer region, Saudi Arabia, and identify predictors and associated factors. A cross-sectional survey using a structured questionnaire following a self-administrative study was conducted across community pharmacies in the Aseer region (Abha, Khamis Mushait, Mahayel, Sarat Abeeda, Ahad-Rufaida, and Bishah). A total of 206 respondents from community pharmacists participated in the study. Over the last six months, 136 respondents (66.0%) suspected pregabalin abuse in community pharmacies; male dominance in pregabalin abusers was also recorded (*n* = 165, 80.1%). Additionally, 40 (19.4%) respondents stated that a prescription was not issued for pregabalin demands. Over half (61.7%) of community pharmacists recorded an increased change in pregabalin abuse compared to the previous year. This is the first study to explore pharmacists’ perceptions in the community of the Aseer region towards customers’ misuse and abuse of pregabalin. Further monitoring and regulations on the prescribing and procurement of pregabalin are needed to avoid abuse.

## 1. Introduction

Prescription drug misuse and abuse have been reported as a global issue [1]. The World Health Organization has clearly defined the rationale of drug use as when “patients receive medications appropriate to their clinical needs, in doses that meet their own individual requirements, for an adequate period of time, and at the lowest cost to them and their community” [2]. Drug misuse is when patients use medications in a way other than that prescribed by a physician [3]. However, the National Institute on Drug Abuse (NIDA) defines the misuse of prescription drugs as “taking a drug in a way or dosage that is not prescribed; taking somebody’s prescription, even though it is for a valid medical reason like pain; or taking a drug to feel euphoric” [4,5]. On the other hand, drug abuse is the use of medications in a way that is inconsistent with legal and medical purposes. Both practices are considered inappropriate uses of medication [3]. Records state that the euphoria which appears as an adverse reaction in approximately 10% of patients is a leading cause of abuse [6]. As per statistics from the United Nations Office on Drugs and Crime, about 5% of adolescents who used drugs at least once in 2015 have reportedly suffered from drug use disorders, numbering 29.5 million in total [3,7]. Drug misuse is an increasing economic threat to public health. For problematic drug use in England and Wales, annual social costs have been assumed to be around GBP 11.961 million or GBP 35.455 per year/per user [8,9]. In Saudi Arabia, there is insufficient information on drug abuse; however, some studies have shown that opioids, alcohol, and cannabis are perhaps the most prevalent drugs abused in treatment centers [10].

Pregabalin is an analog of gamma-aminobutyric acid (GABA), the mammalian neurotransmitter. Pregabalin is structurally similar to gabapentin, which is known as an alpha 2 omega ligand [11]. For neuropathic pain, pregabalin is prescribed as the first-line therapy as well as for chronic pain [12,13]. Pregabalin inhibits the release of neurotransmitters (glutamate, noradrenaline, 5-hydroxytryptamine, dopamine, and substance P) at synapses by binding to the α2δ-subunits of presynaptic voltage-dependent calcium channels. The drug blocks the excitability of the neurons, particularly in the central nervous system (CNS) [14]. These neurons’ blocking actions possibly account for the analgesic, anticonvulsant, anxiolytic, and sleep-modulating activities of pregabalin [14,15,16]. As per Pharma Marketing, net pregabalin (Lyrica^®^) sales worldwide in 2014 were ranked 12th (approximately USD 5.4 billion), with an annual growth rate of almost 12% [17].

Preclinical, clinical, and epidemiological observations have raised the issue of pregabalin abuse. In addition, case reports show that illegal pregabalin use is prevalent in opioid-addicted patients (68%) [17,18]. Pregabalin abuse and dependency were first recorded in 2006 in Italy, Germany, and Turkey [19]. Additionally, pregabalin is approved for use to treat neuropathic pain in Japan from fibromyalgia [20]. Pregabalin abuse has evolved from being a prescription drug to being mishandled, similarly to stimulants (methylphenidate), over the last ten years. Over time, it has become more widely available either through online outlets or on the illegal market [19,21]. To boost the overall psychogenic effect, pregabalin has often been mixed with alcohol, benzodiazepines (BZDs), zopiclone, gabapentin, cannabis, methamphetamine, morphine, amphetamines, LSD, and mephedrone [21]. The use of pregabalin and opiates at the same time has been linked to a substantially increased risk of mortality [19]. When combined with opioids or other CNS depressants, pregabalin’s misuse potential raises concerns regarding greater risks of respiratory failure and death [22]. A study conducted in Jordan on community pharmacist’s experiences of pregabalin misuse and abuse among their customers reveals that most participants (87.4%) reported cases of pregabalin abuse in their pharmacies [23]. Another study conducted in Lebanon reported that pharmacists might need to improve their knowledge concerning tramadol and gabapentinoids (α₂δ ligands) [24]. Pregabalin was deemed a safe and effective medication for pain and helping with sleep in a Chinese study area at doses of 300–450 mg per day [25]. In Saudi Arabia, only a few studies have discussed pharmacists’ awareness of the misuse and abuse of medications. They reported that pharmacy staff should have sufficient knowledge to identify medication abuse or misuse terminology. In addition, pharmacists’ roles in Saudi Arabia should be clearly defined by drug legislators with policy and regulations toward the misuse and abuse of medication [3]. However, a previous study conducted in Saudi Arabia explored the prevalence of the misuse and abuse of pregabalin among healthcare workers [26]. 

Pregabalin was classified as a controlled substance in 2005 (USA, schedule V) and 2017 (Jordan, schedule III) [23]. Therefore, this study was designed to evaluate community pharmacists’ perception in the Aseer region regarding pregabalin abuse and misuse by customers and determine its predictors and associated factors. The research will also focus on their practices regarding the dispensing of such drugs, especially with their high risk of misuse and abuse.

## 2. Materials and Methods

### 2.1. Study Design

This was a cross-sectional, questionnaire-based, self-administered study conducted across community pharmacies in the Aseer region.

### 2.2. Sample Size and Sampling Technique

The questionnaire was randomly distributed to 90 community pharmacies in six major cities in the Aseer region (Abha, Khamis Mushait, Mahayel, Sarat Abeeda, Ahad-Rufaida, and Bishah). Each community pharmacy had 2–3 pharmacists working over two shifts. As per the latest statistics, the number of registered community pharmacists in the Aseer region eligible to answer the study’s survey was 747 pharmacists [27].

Using the sample size calculation website (http://www.raosoft.com/samplesize.html (accessed on 13 November 2020), the number of potential participants would be 252, implying a 95% confidence interval, 5% margin of error, and 50% response distribution. The data collection process was conducted by approaching community pharmacists in the designated cities and providing them with the survey. All survey-related questions and inquiries were answered. The pharmacists were asked to participate in the survey voluntarily. They were not asked about their ID or the location of their pharmacy. All data were kept confidential and treated with a minimal number of persons during data collection and analysis.

### 2.3. Measures

No previous study has been conducted on community pharmacists’ perceptions towards pregabalin misuse and abuse in Saudi Arabia; therefore, the survey was adopted from four previous studies conducted elsewhere [3,23,24,28]. The questionnaire consisted of 21 questions on 2 domains; Domain I: 10 questions related to demographics; Domain II: 11 questions on perception with open and closed questions related to community pharmacists’ perceptions towards pregabalin misuse and abuse. Initially, a pilot study was conducted on 14 faculty members in the College of Pharmacy at King Khalid University to evaluate the survey’s reliability and validity. A self-administered questionnaire was created, and the pilot sample was then validated to ensure its quality and internal reliability. The Cronbach’s alpha factor was determined as 0.81. In addition, three experts working within this field provided advice regarding the process. The pilot study results were not included in the final analysis.

### 2.4. Statistical Analyses

The questionnaires were reviewed for completeness and accuracy, and the data were cleaned, coded, and then entered into SPSS version 20 (IBM Corp., Armonk, NY, USA). The sociodemographic data were represented using descriptive analysis. Categorical variables were reported as frequencies and percentages, and continuous variables were described as means and standard deviations. Two multiple logistic regressions were conducted to investigate the factors that affected the following two dependent variables: (1) the perceived change in pregabalin abuse during the last year; and (2) the strategy used to limit suspected customers’ access to pregabalin products of abuse. Regarding the second model (strategies used to limit the access to pregabalin), any effort to sell pregabalin without a prescription was considered as incorrect behavior, whereas refusing to sell the product was considered correct behavior. A particular independent variable was included in the final multiple logistic regression if it, upon conducting bivariate logistic regression with the previous two dependent factors, resulted in a *p*-value equal to or less than 0.20. Odds ratios and 95% confidence intervals were tabulated and analyzed.

## 3. Results

### 3.1. Demographics of the Respondents

A total of 206 respondents participated in the study. Community pharmacies completed questionnaires in the six major cities in the Aseer region (Abha, Khamis Mushait, Mahayel, Sarat Abeeda, Ahad-Rufaida, and Bishah). The majority of respondent pharmacists were male (*n* = 157, 76.2%), and the respondents’ average age was between 20 and 30 years (*n* = 125, 60.7%). The most common educational backgrounds were found to be a Doctor of Pharmacy or PharmD (*n* = 103, 50%) and a Bachelor of Pharmacy (*n* = 89; 43.2%). Almost half of the participating pharmacists had 1–4 years’ experience (*n* = 97, 47.1%), whereas 30.1% had less than one year of experience (*n* = 49, 32.5%). Additionally, 90.8% of participants’ pharmacies were located in an urban area (*n* = 187), and 88.3% were chain pharmacy types. Most of the pharmacies were located on a main road (*n* = 130, 63.1%). More than half of the pharmacists (*n* = 114, 535.3%) reported that they generally received an incentive for selling drugs. An overview of the respondents is given in Table 1.

### 3.2. Community Pharmacists’ Perceptions toward Customer’s Misuse and Abuse of Pregabalin

Table 2 shows that 80.6% of the pharmacists sold pregabalin with the presentation of prescriptions, whereas 19.4% (*n* = 40) sold the drugs without prescriptions. The majority of respondents (*n* = 136, 66.0%) suspected pregabalin product abuse/misuse in community pharmacies in the last 6 months. Male dominance in the pregabalin-abusers was also reported (*n* = 165, 80.1%). Young adults aged 20–30 years (*n* = 128; 62.1%) were the majority of abusers, compared to those aged <20 years (9.7%) and >40 years (8.7%). The most commonly abused strengths were 75 mg (*n* = 72, 35%) and 150 mg (*n* = 73, 35.4%). It was reported that most abusers had indications of back pain (*n* = 60, 29.1%), followed by neuropathy (*n* = 44, 21.4%), epilepsy (*n* = 39, 18.9%), chronic pain (*n* = 33, 16%), and others (*n* = 30, 14.6%). The majority of those suspected of abusing or misusing pregabalin products were foreigners (non-usual customers of the pharmacy) (*n* = 152, 73.8%).

### 3.3. Perceived Change of Pregabalin Misuse and Abuse

**Figure 1:**
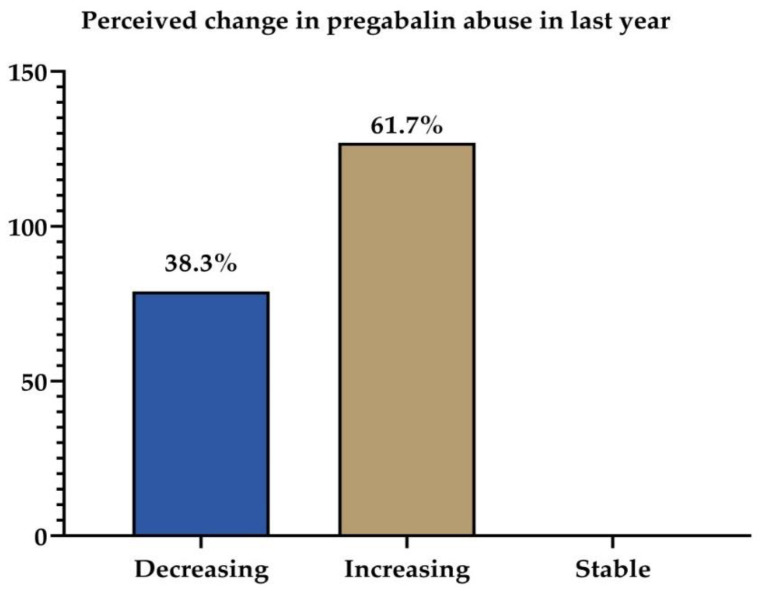
Perceived change of pregabalin misuse and abuse in the Aseer region (*n* = 206).

The majority of the pharmacists who took part in the study noticed an increase (*n* = 127/206, or 61.7%) in the pattern of pregabalin abuse/misuse over last year, as shown in Figure 1. In contrast, a 38.7% decreased pattern was also observed for pregabalin abuse/misuse. The variables that calculated the logistic regression model for the perceived change in pregabalin abuse during the last year are presented in Table 3, and two of them were chosen to be included in the final multiple logistic regression (i.e., pharmacy type and the claimed indication of abusers). The final multiple logistic regression that examined the perceived change in pregabalin abuse during the last year is presented in Table 4. The “chain pharmacy” and “back pain” categories were considered reference or independent variables for pharmacy type and claimed indication of abusers, respectively. The multiple logistic regression indicated that the odds of increasing use of pregabalin in the last year was higher in private pharmacies compared to chain pharmacies (OR = 2.698). Furthermore, the abuser was more likely to have back pain compared to chronic pain (OR = 0.423), neuropathy (OR = 0.774), epilepsy (OR = 0.60), and others (OR = 0.406). However, none of these dependent variables reveal a significant *p*-value.

### 3.4. Strategy to Limit Suspected Customers’ Access to Pregabalin Abuse 

Pharmacists used various techniques to limit consumers’ access to pregabalin abuse, with refusing to sell being the most mentioned in the present study (*n* = 177; 85.9%). However, 20 pharmacists (9.7%) reported that they did nothing about the issue and sold the demanded pregabalin (Table 5). All of these pharmacists were over the age of 30 (*p*-value ≤ 0.001). More than 115 pharmacists (55.8%) reported that the government’s legal restrictions help reduce pregabalin use, although 57 pharmacists (27.7%) did not agree with this statement. Table 6 shows the variables chosen for inclusion in the final logistic regression model used to assess the strategy used to limit suspected customers’ access to pregabalin products of abuse. The final multiple logistic regression is presented in Table 7. Younger pharmacists seemed to express the correct behaviors by not selling pregabalin without a prescription (i.e., pharmacists aged between 31 and 40 years were less likely to avoid selling pregabalin without a prescription (OR = 0.237) compared to pharmacists aged 20–30 years). Additionally, PharmD pharmacists were more likely to avoid selling pregabalin without a prescription (OR = 9.04) compared to pharmacists with a diploma degree.

## 4. Discussion

This study underlined community pharmacists’ perceptions towards the misuse and abuse of pregabalin in the Aseer region of Saudi Arabia. The area studied included six cities: Abha, Khamis Mushait, Mahayel, Sarat Abeeda, Ahad-Rufaida, and Bishah. There has been no such type of study of community pharmacists regarding pregabalin misuse in the Aseer region of Saudi Arabia. However, a study was conducted from July 2017 to July 2018 in the Aseer region among healthcare professionals (physicians, pharmacists, nurses, and paramedical staff) regarding the prevalence of pregabalin abuse. This study among healthcare professionals reported that 42.9% of abusers used pregabalin for stress management, whereas 52% of abusers used it with more than one other drug [26]. In a cross-sectional study in the Eastern Region of Saudi Arabia, 48.9% (*n* = 44) of the respondents stated that the misuse and abuse of drugs in Saudi Arabia was at an alarming level [3]. Pregabalin was listed as a controlled drug in the United States in 2005 (schedule V) and 2017 in Jordan (schedule III) [23]. As for Saudi Arabia, selling pregabalin without prescriptions is restricted, although some reports have found that this still occurs. We also believe that there have been more confirmed cases of pregabalin abuse in community pharmacies.

According to some reports, pharmacists receive inadequate instruction or training in the area of drug abuse. Pharmacy students and pharmacists, in particular, are underprepared to identify, interact with, or manage patients and co-workers who have drug abuse issues [29]. The pharmacy profession’s licensing laws and ethics are designed to protect the public and maintain professional boundaries [30]. Community pharmacists are by far the most responsive healthcare professionals and the first line of support against prescription and non-prescription drug abuse; therefore, it is considered that scheduling the drug and the subsequent strengthening of inspections on its sale in community pharmacies would help minimize this issue [23,31]. In one study, 89.5% of community pharmacists distributed antipsychotic medications based on co-worker’s requests without reviewing the prescriptions [32]. In this study, 19.4% of community pharmacists received a request to sell pregabalin without a prescription. However, according to the survey, 36.4% of the community pharmacists agreed that dispensing drugs without a prescription is vital for the pharmacy’s profits [29]. In this study, 55.3% of community pharmacists reported receiving incentives while selling drugs in general.

More than half of the community pharmacists in our study indicated that they had received suspicious demands for pregabalin in the previous six months, most of which were for strengths of 75 mg or 150 mg and with indications of back pain (29.1%), followed by neuropathy (21.4%), epilepsy (18.9%), and chronic pain (16%). According to a previous survey, pregabalin abusers used the drug for euphoria (28.6%), whereas 42.9% used it for stress management [26]. Furthermore, in this study, males were suspected of abusing pregabalin in 80.1% of cases. This study is similar to a previous study conducted in the Aseer area for healthcare professionals [26]. Several other studies in the literature look at the male gender as a possible cause for addictive behavior [23,33,34]. It also observed that foreign (i.e., non-regular) customers are more likely to be pregabalin product abusers compared to local or usual customers.

The majority of pharmacists reported a growth (61.7%) in pregabalin abuse/misuse patterns over the previous year, although a decrease (38.7%) was noted by some. This increase in pregabalin abuse might be due to the incentives offered by customers for sales, which is also mentioned by Al-Husseini and colleagues [23]. The perceived change in pregabalin abuse during the last year was also associated with the work experience, where it was reported that pharmacists with less than one year of experience were mostly involved. A study showed that only 71% of community pharmacists had received comprehensive instruction on drug misuse/abuse since Pharmacy School graduation [35]. In another survey from the Center on Addiction and Substance Abuse in 2005, only 48% had received training to prevent drug addiction [36]. However, one study showed that most community pharmacists were taught or trained to detect abuse or dependency during a pharmacy bachelor’s degree. However, approximately 85.8% of community pharmacists indicated a desire to receive advanced educational programs in drug abuse [29,30]. Pharmacist participation in community service and substance addiction management studies should be facilitated as well as the inclusion of training or specialization in pharmacy residency programs [30].

Prescription-only medication does not authorize pharmacists to dispense medicine without a prescription written by a doctor, and this is where a pharmacist’s professional and ethical judgment is essential. Some pharmacists might respond by selling large quantities of the products that have been requested [37]. In this study, 9.7% of community pharmacists agreed to sell the requested pregabalin, despite it being an unethical practice to do so. The community pharmacists in the Aseer area studied in this study limited pregabalin product abuse by applying the legal restrictions from the government; 55.8% of the community pharmacists agreed with the above statement. Traditionally, pharmacists have used different strategies, such as refusing to sell such drugs, placing them out of sight, or demanding a medical prescription [38,39]. Therefore, our study results suggested that 85.9% of community pharmacists agreed to refuse the sale to limit suspected customers’ access. However, these techniques are of little use because patients can obtain supplies from other pharmacies [40]. This issue can be reduced if pharmacists network with each other more often; if a suspected abuser is identified to other local pharmacies, they can be notified systematically by linking all pharmacies on a national level by an electronic system. This form of model requires community pharmacist training as well as increased collaboration with healthcare professionals [40,41].

This study has a few limitations, which are: (1) Saudi Arabia’s health authorities have restricted the sale of pregabalin without a prescription [42], but some people can still obtain the drug; (2) The data presented in this study were focused on community pharmacists’ perceptions towards day-to-day events, which are utterly personal and only represented single perspectives. As a result, a critical reflection in the pharmacy-based analysis is more reliable in this context; (3) The questionnaire was answered by community pharmacists in Saudi Arabia’s Aseer area, which is not representative of Saudi Arabia overall; and (4) The sample size was relatively small. As a result, the current study’s findings can only reflect the situation in the Aseer area. Future research should include a higher proportion of community pharmacies from various regions across Saudi Arabia.

## 5. Conclusions

This study provides a basic representation of community pharmacists’ attitudes, awareness, and opinions in the Aseer region of Saudi Arabia, addressing pregabalin abuse. According to participating pharmacists, pregabalin could be abused, with young males being the most likely pregabalin abusers. A substantial number of pregabalin demands were not followed by a prescription. There was a perceived change in pregabalin misuse and abuse in the Aseer region over the previous year. These results highlight the importance of developing better pharmacy-based programs to increase drug prescribers’ (e.g., physicians, neurosurgeons, and pharmacists) awareness regarding the potential misuse of pregabalin. Further monitoring and regulations on the prescribing and procurement of pregabalin are needed to avoid the abuse, and a strict policy for pharmacists regarding drug misuse and abuse is also needed in Saudi Arabia.

## Figures and Tables

**Table 1:** Demographics of the respondents.

Items	Sample (*n* = 206)	Percentage (%)
Gender		
Male	157	76.2
Female	49	23.8
Age		
20–30 years	125	60.7
31–40 years	61	29.6
41–50 years	18	8.7
Over 50 years	2	1.0
Education Level		
Pharmacy diploma	7	3.4
Bachelor in Pharmacy	89	43.2
Doctor of Pharmacy (PharmD)	103	50.0
Postgraduate	7	3.4
Years of Experience		
Less than one year	62	30.1
1–4 years	97	47.1
5–10 years	34	16.5
More than 10 years	13	6.3
Pharmacy Area		
Rural	19	9.2
Urban	187	90.8
Pharmacy Type		
Chain	182	88.3
Private	24	11.7
Pharmacy Location		
Main street	130	63.1
By street (village/district pharmacy)	52	25.2
Inside shopping center	24	11.7
Work-Shift Time		
Morning shift	74	35.9
Mid-day shift	80	38.8
Night shift	52	25.2
Do you receive incentives when you sell drugs in general? (Yes)	114	55.3

**Table 2:** Community pharmacists’ perceptions towards customer’s misuse and abuse of pregabalin.

Items	Subgroups	Sample (*n* = 206)	Percentage (%)
Do you receive requests to sell pregabalin with a prescription?	No, not at all	40	19.4
	Yes, sometimes	62	30.1
	Yes, usually	74	35.9
	Yes, always	30	14.6
In the last 6 months, have you received a request to sell pregabalin to someone who misused or abused them?	Yes	136	66.0
Most of the time, what is the gender of assumed pregabalin abusers who come to your pharmacy?	Male	165	80.1
Most of the time, what is the age of assumed pregabalin abusers who come to your pharmacy?	Less than 20 years	20	9.7
	20 to 30 years	128	62.1
	31 to 40 years	40	19.4
	More than 40 years	18	8.7
What is the most requested dose of pregabalin from assumed abusers who come to your pharmacy?	25 mg	6	2.9
	50 mg	23	11.2
	75 mg	72	35.0
	150 mg	73	35.4
	300 mg	32	15.5
What was indication that the assumed abusers of pregabalin claimed they had when they requested pregabalin?	Back pain	60	29.1
	Chronic pain (other than back pain)	33	16.0
	Neuropathy	44	21.4
	Epilepsy	39	18.9
	Other	30	14.6
Usually, who are the assumed abusers of pregabalin who come to your pharmacy?	Non-regular/foreign customers of the pharmacy	152	73.8
	Usual customers at the pharmacy	54	26.2

**Table 3:** Bivariate logistic regression analysis for the perceived change in pregabalin abuse during the last year.

Variable	Wald Test	*p*-Value
Gender	0.071	0.789
Age	0.275	0.675
Educational level	0.002	0.966
Work experience	6.408	0.421
Pharmacy area	0.125	0.724
Pharmacy type	3.325	0.068
Pharmacy location	0.916	0.339
Work shift	0.434	0.510
Incentives to sell drugs	0.007	0.935
Gender of abusers	0.949	0.330
Age of abusers	1.099	0.295
Claimed indication of abusers	2.176	0.140
Strategy used by you to limit suspected customers’ access to pregabalin products of abuse	0.002	0.961

**Table 4:** Multiple logistic regression examining potential factors affecting the perceived change in pregabalin abuse during the last year (if OR is more than 1, this means that the misuse of pregabalin was increased in comparison to the last year).

Variable	OR (95% CI)	*p*-Value
Pharmacy type		
Chain Pharmacy	Reference	
Private pharmacy	2.698 (0.939–7.751)	0.651
Claimed indication of abusers		
Back pain	Reference	
Chronic pain (other than back pain)	0.423 (0.174–1.029)	0.058
Neuropathy	0.774 (0.326–1.834)	0.56
Epilepsy	0.6 (0.257–1.401)	0.237
Other	0.406 (0.163–1.011)	0.053

**Table 5:** Strategy used to limit suspected abusers’ access to pregabalin in the Aseer region (*n* = 206).

Items	Subgroups	Sample (*n* = 206)	Percentage (%)
What is the strategy used by you to limit suspected abusers’ access to pregabalin products?	Do nothing. Just sell the product	20	9.7
	Selling smaller amount than requested	9	4.4
	Refusal of sale	177	85.9
Do you think that the legal restrictions applied by the government help in reducing pregabalin abuse?	No	57	27.7
	Yes	115	55.8
	I don’t know	34	16.5

**Table 6:** Bivariate logistic regression analysis for the strategies used to limit suspected abusers’ access to pregabalin products.

Variable	Wald Test	*p* Value
Gender	5.161	0.023
Age	11.47	<0.001
Educational level	3.080	0.079
Work experience	2.170	0.141
Pharmacy area	2.549	0.117
Pharmacy type	1.005	0.316
Pharmacy location	1.263	0.261
Work shift	0.638	0.424
Incentives to sell drugs	0.615	0.433
Gender of abusers	8.956	0.003
Age of abusers	5.656	0.017
Claimed indication of abusers	1.035	0.905
Usually, the assumed abusers of pregabalin who come to your pharmacy are usual or foreigner customers	22.158	<0.001

**Table 7:** Multiple logistic regression examining potential factors affecting the strategy used to limit suspected abusers’ access to pregabalin products (if OR is more than 1, this means that the odds of correct behavior, i.e., not selling pregabalin, was higher in this group compared to the reference).

Variable	OR (95% CI)	*p*-Value
Gender		
Female	Reference	
Male	0.123 (0.015–1.005)	0.051
Age of the Pharmacist		
20–30 years	Reference	
31–40 years	0.237 (0.079–0.712)	0.01
41–50 years	0.224 (0.057–0.874)	0.031
Over 50 years	0.175 (0.003–10.281)	0.401
Education level		
Pharmacy diploma	Reference	
Bachelor in Pharmacy	5.322 (0.926–30.583)	0.061
Doctor of pharmacy (PharmD)	9.04 (1.513–53.999)	0.016
Postgraduate	1.19 (0.116–12.169)	0.884
Work Experience		
Less than one year	Reference	
1–4 years	2.901 (0.886–9.497)	0.078
5–10 years	1.629 (0.436–6.091)	0.468
More than 10 years	6.002 (0.708–50.904)	0.1
Pharmacy Area		
Rural	Reference	
Urban	1.526 (0.406–5.738)	0.532
Gender of Abusers		
Female	Reference	
Male	2.319 (0.829–6.486)	0.109
Age of Abusers		
Under 20 years	Reference	
20 to 30 years	3.85 (0.799–18.551)	0.093
31 to 40 years	9.625 (2.828–32.761)	<0.001
Over 40 years	21.962 (3.545–136.042) 0.001	<0.001
Types of Customers		
Non-usual/Foreigner customers of the pharmacy	Reference	
Usual customers at the pharmacy	5.394 (1.967–14.794)	0.001

## Data Availability

Data are available upon request.
